# Redescription of *Protoopalina pingi* Nie, 1935 inhabiting the recta of *Hylarana guentheri* and *Pelophylax nigromaculatus* in China

**DOI:** 10.1051/parasite/2014021

**Published:** 2014-09-12

**Authors:** Weidong Li, Chong Wang, Feng Huang, Ming Li, Frank Nilsen, Huiyu Liu, Jianlong Xu

**Affiliations:** 1 Hubei Key Laboratory of Animal Nutrition and Feed Science, Wuhan Polytechnic University Wuhan 430023 China; 2 Institute of Hydroecology, Ministry of Water Resources & Chinese Academy of Sciences Wuhan 430079 China; 3 Sea Lice Research Centre, Department of Biology, University of Bergen Bergen 5020 Norway; 4 Hubei Collaborative Innovation Centre for Freshwater Aquaculture Wuhan 430070 China

**Keywords:** *Protoopalina pingi*, flagellate, frog, *Hylarana guentheri*, *Pelophylax nigromaculatus*

## Abstract

A redescription of *Protoopalina pingi* Nie, 1935 is presented in this paper to complete Nie’s description at both light and scanning electron microscope levels. These organisms were collected from the recta of the frogs *Hylarana guentheri* Boulenger, 1882 and *Pelophylax nigromaculatus* Hallowell, 1861 from Jialing River, Sichuan Province and Honghu Lake, Hubei Province, respectively, in China. This is the first record of its occurrence in *H. guentheri* and *P. nigromaculatus*. The body of *P. pingi* is elongated and somewhat spindle-like in shape, slightly narrowed and bluntly rounded at the anterior extremity, while the posterior end is tapering or sharply pointed. The body surface is thickly flagellated, with the caudal tip being barren. The falx, located at the margin of the anterior end, is composed of a narrow band of kinetosomes. Four round or oval-shaped nuclei, usually arranged in a straight line, are situated in the middle region of the body. Comparisons are made between *P. pingi* and its congeners.

## Abstract (fr)

Une redescription de *Protoopalina pingi* Nie, 1935 est présentée dans cet article, pour compléter la description de Nie par la microscopie photonique et électronique. Ces organismes ont été recueillis à partir du rectum des grenouilles *Hylarana guentheri* et *Pelophylax nigromaculatus*, respectivement de la rivière Jialing dans la province du Sichuan et du Lac Honghu dans la province du Hubei, en Chine. C’est la première mention chez *H. guentheri* et *P. nigromaculatus*. Le corps de *P. pingi* est allongé et fuselé, légèrement rétréci et grossièrement arrondi à l’extrémité antérieure, tandis que l’extrémité postérieure est effilée ou très pointue. La surface du corps est fortement flagellée, mais la pointe caudale est nue. La faux, situé sur la marge de l’extrémité antérieure, est composée d’une bande étroite de cinétosomes. Quatre noyaux ronds ou ovales, généralement disposés le long d’une ligne droite, sont situés dans la région médiane du corps. Des comparaisons sont faites entre *P. pingi* et ses congénères.

## Introduction

Opalinids, originally discovered by Leeuwenhoek in 1683 [7], are multinuclear, mouthless, osmotrophic flagellated protozoa. They live as commensals in the digestive tracts of different poikilothermic vertebrates, especially anuran amphibians [15]. The opalinids were for a long time regarded as the astomatous (no cytostome) ciliates because of their superficial similarities with the ciliates and were given the status “protociliates” as opposed to “euciliates” since the monomorphic nuclei, in contrast to dimorphic nuclei, were suggested to be an ancestral state of ciliates [1, 13, 14, 23]. Then the hypothesis of opalinid-ciliate affinity was abandoned since other characteristics, such as the structure of the nucleus, the mode of cell division and the reproductive cycle, differed remarkably from those of ciliates and these organisms were deemed to be either an isolated taxon in the phylum Zooflagellata or were treated as a separate phylum: Opalinata [3, 4, 8, 24]. Now, it has been convincingly shown that opalinids belong to heterokonts as a sister group to *Proteromonas* within the order Slopalinida based on detailed ultrastructural study and believable phylogenetic analyses [2, 6, 10, 11, 16, 17, 19, 20, 22].

The family Opalinidae can be separated into two subfamilies, Protoopalininae and Opalininae, based on the shape of the cell body and the number of nuclei. The subfamily Opalininae is comprised of the genera *Cepedea* Metcalf, 1920 and *Opalina* Duskinje and Valentin, 1835, while the subfamily Protoopalininae contains the genera *Protoopalina* Metcalf, 1918 and *Zelleriella* Metcalf, 1920.

*Protoopalina* is the most common genus of opalinids inhabiting anuran amphibians [5, 21]. It was established by Metcalf in 1918. Thereafter, many new species of *Protoopalina* have been found from the anuran amphibians. *Protoopalina pingi* was first discovered and named by Nie in 1935 from the intestines of *Rana plancyi* Lataste, 1880 [18]. Although discovered more than 70 years ago, many biological aspects of *P. pingi* are still unknown. After simple morphological information, no further data about this opalinid have been reported. The previous morphological data, however, are incomplete, and some descriptions of important taxonomic structures also need revision. This study adds to Nie’s description and attempts to contribute to the knowledge of this genus.

## Materials and methods

Host frogs, including 256 *H. guentheri* and 104 *P. nigromaculatus*, were captured from Jialing River in Pengan county (31°15′–31°29′ N; 106°12′–106°25′ E), Sichuan Province, China, in August 2011 and Honghu Lake (29°40′–29°58′ N; 113°12′–113°26′ E), Hubei Province, China, in June 2012, respectively. They were transported alive to the laboratory for further examination. We obtained the permits allowing us to capture and sacrifice these specimens. All frog samples were dissected, with the intestines and recta being opened and put into Petri dishes for examination. Then a 0.65% saline solution was added to the samples and we waited for a few minutes to allow *P. pingi* to swim out of the gut contents. The flagellates were collected with a Pasteur micropipette and washed twice in distilled water.

For light microscopy, individuals were observed, measured and photographed in vivo using both bright-field and differential interference contrast microscopy (Zeiss Axioplan 2 imaging and Axiophot 2, Oberkochen, Germany). The remaining specimens were placed directly on coverslips, fixed in a saturated HgCl_2_ solution and stained with Heidenhain’s haematoxylin and a 1% Protargol solution. All measurements are in micrometres.

For scanning electron microscopy (SEM), the washed specimens were fixed in 2.5% glutaraldehyde in 0.2M phosphate buffered saline (PBS, pH 7.4) on a clean glass slide (1 cm × 1 cm), previously treated with 0.1% poly-L-Lysin and dried completely in air at room temperature (RT). After being washed with PBS three times, they were post-fixed in 1% osmium tetroxide at 4 °C for 1 h, followed by serial dehydration in acetone and critical point drying using a HCP-2 critical point dryer (Hitachi Science Systems, Ibaraki, Japan). Then the glass slide was mounted on an aluminium stub using double-sided adhesive tape and sputter-coated with a thin layer of gold in an IB-3 ion coater (Eiko Engineering, Ibaraki, Japan) before observing and photographing with a Quanta 200 SEM (FEI, Amsterdam, Netherlands).

## Results

One hundred and thirty-five of the 256 *H. guentheri* examined and 42 of the 104 *P. nigromaculatus* examined were found to be infected with *P. pingi*. Large numbers of *P. pingi* were found in the recta of all frog hosts that contained them.

### *Protoopalina pingi* Nie, 1935

Host: *Hylarana guentheri* Boulenger, 1882 and *Pelophylax nigromaculatus* Hallowell, 1861.

Prevalence: Total 135 (52.7%) out of 256 *H. guentheri* and 42 (40.4%) of 104 *P. nigromaculatus* were infected with this opalinid, respectively.

Habitat: Rectum.

Locality: Jialing River, in Pengan county, Sichuan Province, China; Honghu Lake, in Honghu City, Hubei Province, China.

Deposited specimens: Slides 2012W001-003 of Heidenhain’s haematoxylin-stained specimens, and slides 2012W004-010 of Protargol-stained specimens have been deposited in Hubei Key Laboratory of Animal Nutrition and Feed Science, Wuhan Polytechnic University, China.

**Figure 1.**
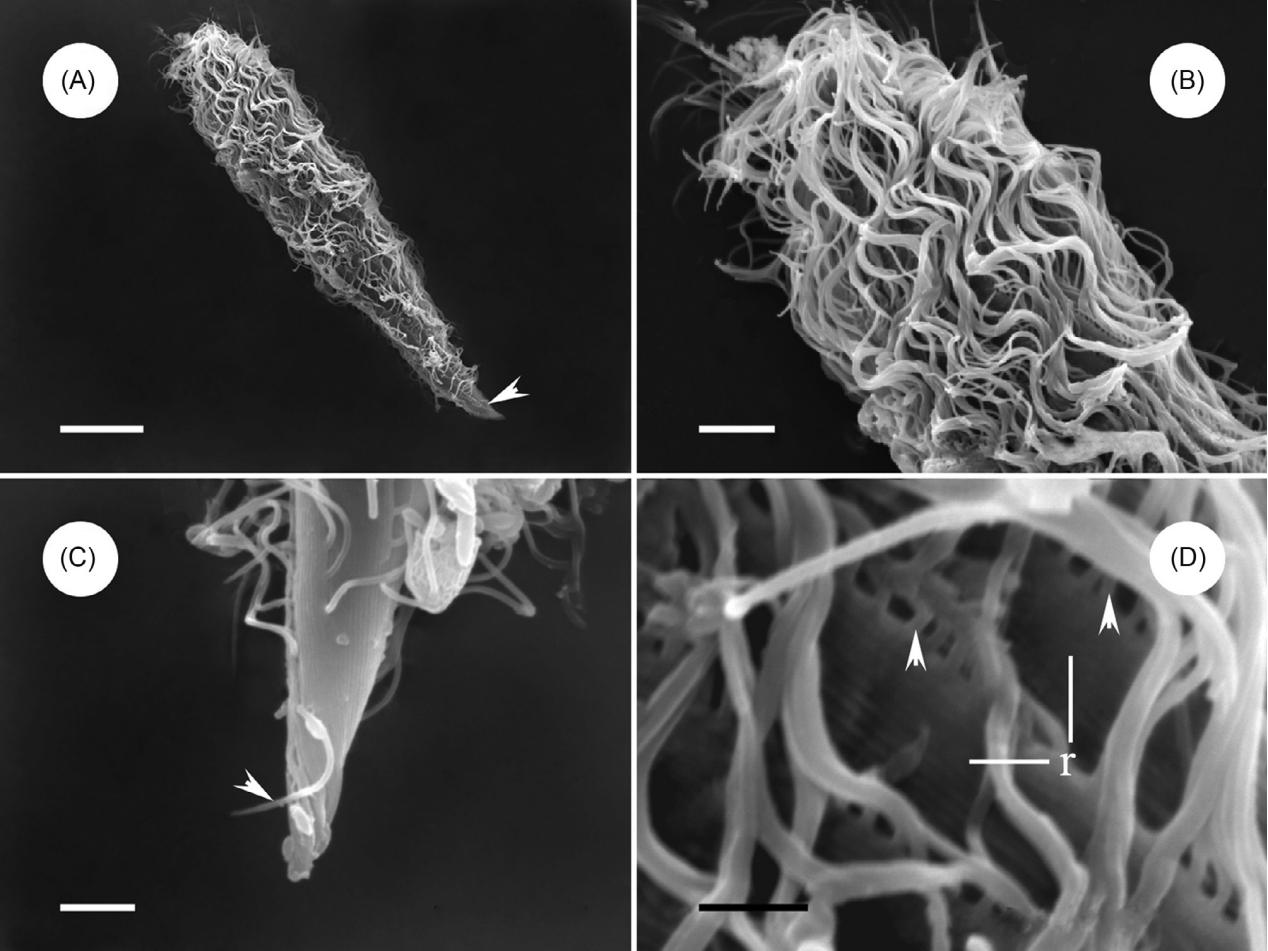
Scanning electron microscope images of *Protoopalina pingi* Nie, 1935. (A) Overview of *P. pingi*, showing many fused flagella over the body. *Scale bar* = 20 μm. (B) Anterior end of *P. pingi*, showing the densely flagellated body surface. *Scale bar* = 5 μm. (C) Caudal tip of *P. pingi*, showing the flagella (arrowhead) in the region barren of flagella. *Scale bar* = 2.5 μm. (D) The flagella are arranged in the ridge, showing the proximal ends of the flagella (arrowhead) and ridge (*r*). *Scale bar* = 1.5 μm.

**Figure 2.**
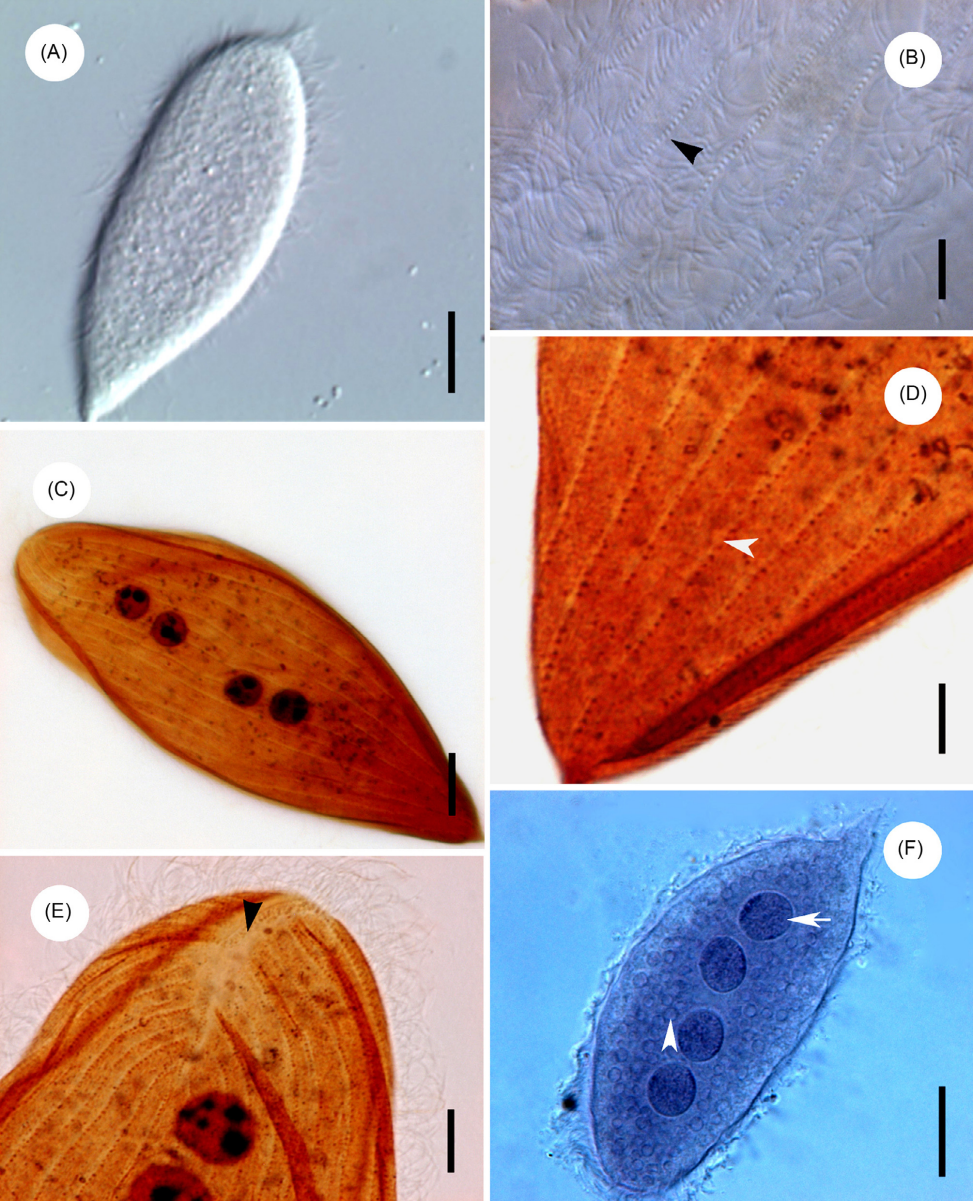
Light microscope images of *Protoopalina pingi* Nie, 1935. (A) Living specimens, showing the normal trophozoites of *P. pingi*. *Scale bar* = 20 μm. (B) Living specimens, showing the flagella covering the body (arrowhead). *Scale bar* = 5 μm. (C) Specimens stained with Protargol, showing the somatic kineties and the nuclei with distributed nucleoli. *Scale bar* = 10 μm. (D) Specimens stained with Protargol, showing the somatic kineties in the posterior extremity (arrowhead). *Scale bar* = 5 μm. (E) Specimens stained with Protargol, showing the falx region in the anterior extremity (arrowhead). *Scale bar* = 5 μm. (F) Specimens stained with Heidenhain’s haematoxylin, showing the nuclei (arrow) and the corpuscles of uneven size (arrowhead). *Scale bar* = 20 μm.

**Figure 3.**
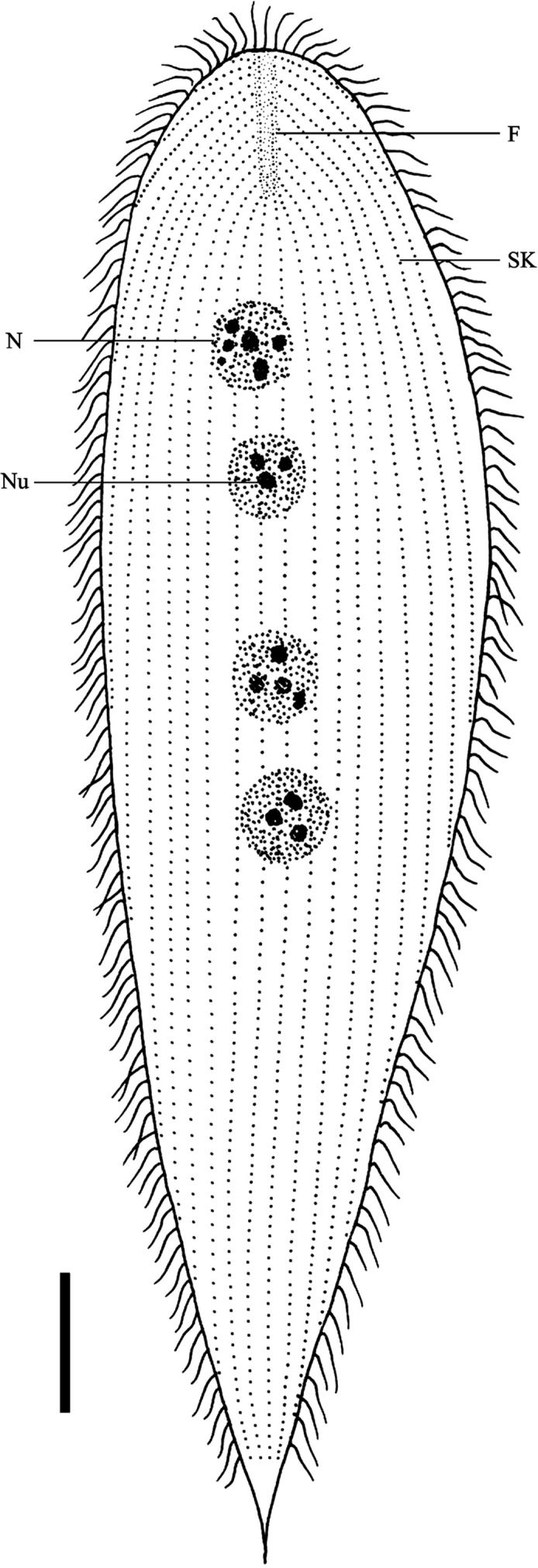
Schematic drawing of *Protoopalina pingi* Nie, 1935, showing the general form and structures: falx (F), nucleus (N), nucleolus (Nu) and somatic kineties (SK). *Scale bar* = 10 μm.

Description: The body is elongated and somewhat spindle-like in shape, slightly narrowed and bluntly rounded at the anterior extremity, while the posterior end is tapering or sharply pointed (Figs. 1A and 2A). The body length is 115.9 μm (93.6–144.0 μm, *n* = 20) and the width 31.1 μm (21.6–48.4 μm, *n* = 20) in vivo. The ratio of length to width is about 4:1. The body surface is thickly flagellated (Figs. 1A, B and 2B) with the caudal tip being barren (Figs. 1A, C). The flagella are arranged in the ridge running parallel to the longitudinal axis, most of which are fused into groups of two or three in our specimens (Figs. 1B, D). All the somatic kineties converge on the falx, anteriorly, and many extend to the posterior extremity (Figs. 2C, D and 3) and number 18–29 in total. The falx, composed of a narrow band of kinetosomes, can be observed in Protargol-impregnated specimens at a higher magnification. It is located at the margin of the anterior pole and extends to both dorsal and ventral sides (Figs. 2E and 3). Four round or oval-shaped nuclei are situated in the middle region of the body, usually with many nucleoli distributed within the karyoplasm (Figs. 2C, F and 3). Normally, the nuclei are arranged in a straight line running parallel to the longitudinal axis (Figs. 2C, F, and 3). The nuclei range in length from an average of 10.8 μm (7.0–15.0 μm, *n* = 15) and in width 8.0 μm (5.0–11.5 μm, *n* = 15) in Protargol specimens. Many apparent corpuscles of uneven size can be observed over the cytoplasm (Fig. 2F).

Data for measurements related to morphometric characteristics are given in Table 1.

**Table 1.** Morphometric light microscopic characterisation of *P. pingi*.

Character	Min	Max	Mean	*SD*	*CV* (%)	*N*
Body length, in vivo	93.6	144.0	115.9	13.6	11.7	20
Body width, in vivo	21.6	48.4	31.1	4.7	16.3	20
Body length, Protargol	72.0	110.4	89.5	10.3	11.6	15
Body width, Protargol	14.4	36.4	23.1	3.7	18.5	15
Nucleus length, Protargol	7.0	15.0	10.8	2.1	19.8	15
Nucleus width, Protargol	5.0	11.5	8.0	1.5	18.7	15
Number of total somatic kineties	18	29	23.6	3.3	13.9	10

Measurements in μm; Min = minimum, Max = maximum, Mean = arithmetic mean, *SD* = standard deviation, *CV* = coefficient of variation, *N* = number of individuals investigated.

## Discussion

As mentioned above, *P. pingi* was first discovered and named by Nie from the intestines of *Rana plancyi* [18]. This is the first record of its occurrence in the recta of *H. guentheri* and *P. nigromaculatus*. The opalinids examined in the present study appear slightly bigger than Nie’s type specimens, since he gave ranges of 55–160 μm by 12.5–57 μm in length and width. Also, the caudal tip of *P. pingi* is barren of flagella according to our SEM observation, which is different from that described by Nie [18]. He stated in his paper that “the cilia covering the entire surface of the body are of moderate size and closely arranged in many oblique or longitudinal rows”. He was likely limited in his views of these flagellates due to the limits of staining techniques and observing equipment in his time. Due to the absence of other morphological data, it is impossible to compare our results with Nie’s records.

With respect to the body outline and nucleus shape, *P. pingi* resembles *P. caudata michyla* [18], *P. quadrinucleata* [12], *P. heleophrynes* [5] and *P. pomacantha* [9]. All these five species have a slightly bent body, pointed posterior end and a blunt anterior extremity with a small falx. However, *P. pingi* can be discriminated distinctly from the others considering the number of nuclei. *P. caudata michyla*, *P. heleophrynes* and *P. pomacantha* have two nuclei, while *P. quadrinucleata* has 1–8. Furthermore, *P. caudata michyla* discovered in *Microhyla ornata* has relatively longer and wider body dimensions (120–290 × 40–70 vs. 93.6–144 × 21.6–48.4 μm) and larger nuclei than *P. pingi* (15–23 × 15–18.8 vs. 7–15 × 5–11.5 μm). *P. quadrinucleata*, inhabiting *Rana guentheri*, is smaller than our present opalinids for body size (58–109 × 10–18 vs. 93.6–144 × 21.6–48.4 μm). *P. heleophrynes* reported in tadpoles of *Heleophryne rosei* also has relatively smaller body dimensions than *P. pingi* (21–54 × 5.7–12 vs. 93.6–144 × 21.6–48.4 μm) in this paper. *P. pomacantha* found in the rectum of Angelfishes most resembles *P. pingi* considering the body size (157.2 × 28.3 vs. 93.6–144 × 21.6–48.4 μm), and the phenomenon that both of their caudal tips are barren of flagella. Morphological comparison among *P. pingi* and other similar species of *Protoopalina* are presented in Table 2.

**Table 2.** Morphological comparison among *P. pingi* and other similar species of *Protoopalina*.

Species	*BL*	*BW*	*N*_*n*_	*NL*	*NW*	*N*_*s*_	Source of data
*P. pingi*	93.6–144.0	21.6–48.4	4	7–15	5–11.5	18–29	Present paper
*P. caudata michyla*	120–290	40–70	2	15–23	15–18.8	–	Nie (1935) [18]
*P. quadrinucleata*	58–109	10–18	1–8	–	–	–	Lu (1945) [12]
*P. heleophrynes*	21–54	5.7–12	2	–	–	–	Delvinguier et al. (1995) [5]
*P. pomacantha*	157.2	28.3	2	14.6	7.7	26.3	Grim et al. (2000) [9]

Measurement in μm; *BL* = Body length, *BW* = Body width, *N*_*n*_ = Number of nuclei, *NL* = Nucleus length, *NW* = Nucleus width, *N*_s_ = Number of total somatic kineties.

In conclusion, based on general morphological characteristics, *P. pingi* is recorded and redescribed in detail from *H. guentheri* and *P. nigromaculatus*. Future collections will be made at different stages of the hosts’ life cycles to determine if the trophonts always have four nuclei instead of the two usually found in *Protoopalina*, to determine if cysts are formed, to study its possible “infection” routes and further assess the host specificity.
